# Sensing Lanthanide Metal Content in Biological Tissues with Magnetic Resonance Spectroscopy

**DOI:** 10.3390/s131013732

**Published:** 2013-10-11

**Authors:** Dina V. Hingorani, Sandra I. Gonzalez, Jessica F. Li, Mark D. Pagel

**Affiliations:** 1 Department of Chemistry and Biochemistry, University of Arizona, Tucson, AZ 85721, USA; E-Mail: dinah@email.arizona.edu; 2 University of Arizona Cancer Center, Tucson, AZ 85724-5013, USA; E-Mail: jfli@email.arizona.edu; 3 Department of Biomedical Engineering, University of Arizona, Tucson, AZ 85721, USA; E-Mail: sigonzal@email.arizona.edu; 4 Department of Medical Imaging, University of Arizona, Tucson, AZ 85721, USA

**Keywords:** lanthanide metals, bulk magnetic susceptibility, magnetic resonance spectroscopy, concentration measurements

## Abstract

The development and validation of MRI contrast agents consisting of a lanthanide chelate often requires a determination of the concentration of the agent in *ex vivo* tissue. We have developed a protocol that uses 70% nitric acid to completely digest tissue samples that contain Gd(III), Dy(III), Tm(III), Eu(III), or Yb(III) ions, or the MRI contrast agent gadodiamide. NMR spectroscopy of coaxial tubes containing a digested sample and a separate control solution of nitric acid was used to rapidly and easily measure the bulk magnetic susceptibility (BMS) shift caused by each lanthanide ion and gadodiamide. Each BMS shift was shown to be linearly correlated with the concentration of each lanthanide ion and gadodiamide in the 70% nitric acid solution and in digested rat kidney and liver tissues. These concentration measurements had outstanding precision, and also had good accuracy for concentrations ≥10 mM for Tm(III) Eu(III), and Yb(III), and ≥3 mM for Gd(III), gadodiamide, and Dy(III). Improved sample handling methods are needed to improve measurement accuracy for samples with lower concentrations.

## 1. Introduction

MRI contrast agents that consist of Gd(III) chelates are often used to enhance the image contrast of anatomical features during clinical diagnoses. Examples include the identification cerebral lesions during neuroimaging [1], the localization of ischemia and acutely infarcted myocardium during cardiac imaging [2], and the diagnoses of solid tumor morphology during oncological imaging [3]. These MRI contrast agents are also used to assess tissue function, including the tracking of vascular flow for large arteries and veins using MRI angiography [4], and the evaluation of vascular perfusion and permeability in capillary networks using Dynamic Contrast Enhancement MRI [5]. Gd(III) and Dy(III) chelates have been used for similar diagnoses of animal models [6–8].

Lanthanide chelates have also been used for molecular imaging. Gd(III) chelates have been used to detect enzyme activities, metabolites, ions, pH, and temperature by monitoring changes in T1- or T2-relaxation [9]. Dy(III), Tm(III), Eu(III) and Yb(III) chelates have also been developed for molecular imaging, primarily through the mechanism of Chemical Exchange Saturation Transfer (CEST) [10]. These lanthanide chelates contain a labile proton that can be selectively saturated, which reduces the coherent MR signal of the proton. The labile proton of the agent can exchange with water, which transfers the saturation to water and effectively reduces the MR signal of water. The magnitude of this change in MR water signal is sensitive to the chemical exchange rate of the CEST agent, which can be modulated by enzymatic catalysis of the agent's chemical structure, a change in temperature or pH that directly affect the kinetic rates of chemical exchange processes, or binding interactions with metabolites and ions [9].

Clinical translation of MRI contrast agents that consist of lanthanide chelates requires evaluations of biodistributions to assess potential toxicities. In particular, slow clearance from the body can lead to extended retention and subsequent dissociation of a lanthanide ion from the organic chelator, which can cause Nephrogenic Systemic Fibrosis [11]. *Ex vivo* measurements of lanthanide chelate concentrations in various tissues of animal models are often one of the first steps of toxicological assessments. In addition, molecular imaging studies of animal models require the accurate measurement of tissue concentrations of MRI contrast agents to validate imaging results. However, T1 relaxation caused by a Gd(III) chelate, and CEST generated from other lanthanide chelates, are dependent on multiple physico-chemical characteristics, so that converting T1-weighted MR images or CEST MRI results to a measurement of contrast agent concentration is not necessarily accurate [12,13]. Therefore, *ex vivo* validation of contrast agent concentrations can improve *in vivo* studies with MRI contrast agents.

Two methods are currently used to measure concentration of lanthanide chelates in *ex vivo* tissues. Inductively coupled plasma mass spectrometry (ICP-MS) provides outstanding measurement accuracy of concentrations of lanthanide ions as low as 1 ng/mL [14]. However, ICP-MS is relatively expensive, has limited availability at many research institutions, requires frequent calibration with known samples, and requires careful sample preparation and handling. A colorimetric test with xylenol orange, methyl thymol blue, or arsenazo dye can also be used to measure concentration of lanthanide ions as low as 8–50 μM depending on the pH and temperature of the sample [15–17]. These spectrophotometric methods are popular because spectrophotometers are relatively inexpensive and available at mot research institutions. Yet colorimetric analyses require samples that are optically transparent except for the dye, which severely limits the ability to assess tissue samples for lanthanide chelate concentration.

The concentrations of lanthanide chelates have also been measured by employing the Evans method with a NMR spectrometer [18]. Paramagnetic lanthanide ions have an effective magnetic moment, *μ_eff_*, that can cause a bulk magnetic susceptibility (BMS) shift of the solvent [19]. This shift in MR frequency can be compared to the shift of a solvent without the lanthanide ion, which is directly related to the concentration of the ion, [Ln] (Equation (1)) [20]. In this relationship, T represents temperature and s is set to 1/3, −1/6, or 0 for a sample geometry that consists of a cylinder parallel to the main magnetic field, a cylinder perpendicular to the main field, or a sphere, respectively [21,22]. This relationship assumes that the measurement temperature is above the Curie-Weiss temperature and that the diamagnetic and the hyperfine shifts are negligible relative to the BMS shift, which is a good assumption for all paramagnetic lanthanide ions. Equation (1) also assumes that direct interactions between paramagnetic lanthanide chelates have negligible influence on the BMS shift, which has been shown to be a good assumption at practical concentrations [23]:

(1) $$\Delta_{\chi }=\frac{1558s[Ln]\mu_{\text{eff}}^{2}}{T}$$

We investigated whether a protocol could be developed that can measure the BMS shift with NMR spectroscopy to accurately measure lanthanide ion concentrations in *ex vivo* tissues of animal models. We evaluated the accuracies and precisions of measuring the concentrations of Gd(III), Dy(III), Tm(III), Eu(III), and Yb(III) ions and a Gd(III) chelate in solutions of 70% nitric acid. We tested conditions of the NMR spectrometer and sample that may affect the concentration measurement. After establishing correlations for these ions, we then performed similar evaluations with *ex vivo* rat liver and kidney tissues that were complete digested in 70% nitric acid. Our results establish a protocol and analytical criteria for measuring *ex vivo* concentrations of lanthanide-based MRI concentrations using the BMS shift.

## 2. Experimental Section

TmCl_3_ hydrate was acquired from Strem Chemicals Inc. (Newburyport, MA, USA). EuCl_3_, YbCl_3_, DyCl_3_, GdCl_3_ were obtained from Acros Organics (Fair Lawn, NJ, USA). Bovine serum albumin and NaCl were purchased from Thermo Fisher Scientific Inc. (Waltham, MA, USA). ACS-grade, 68%–70% nitric acid was purchased from Mallinckrodt Chemical Inc. (St. Louis, MO, USA). Gadodimide (Omniscan™, GE Healthcare Inc., Waukesha, WI, USA) was obtained from the Department of Radiology at Case Western Reserve University (Cleveland, OH, USA). Rat liver and kidney tissues were provided by the Small Animal Medical Imaging Services of the University of Arizona (Tucson, AZ, USA).

Stock solutions were prepared at 5 mM for GdCl_3_, 10 mM for DyCl_3_, and 50 mM for TmCl_3_, EuCl_3_, and YbCl_3_ using distilled water. The concentration of each stock solution was verified with ICP-MS (Department of Geosciences, University of Arizona). A stock solution of gadodiamide was prepared at 5 mM concentration based on the concentration of the clinical product. A series of concentrations ranging between 0.1–5.0 mM for GdCl_3_ and gadodiamide, 0.5–10 mM for DyCl_3_, and 1.0–50.0 mM for TmCl_3_, EuCl_3_, and YbCl_3_ were then created by serially diluting each stock with distilled water. To maintain accuracy, solutions with concentrations less that 5 mM were created with a final volume of 5 mL. Solutions with higher concentrations were created with a final volume of 1 mL. These samples were then frozen in liquid nitrogen and lyophilized for 24–48 h depending on the sample volume. Concentrated nitric acid was then added to the dried sample, using a volume of 5 mL for dilute samples and 1 mL for concentrated samples.

To test the effect of salt, samples at 15 mM TmCl_3_ or 5 mM GdCl_3_ were prepared with NaCl concentrations ranging from 40 mOsm/L to 300 mOsm/L, using a 1 Osm/L stock solution of NaCl and the TmCl_3_ and GdCl_3_ stock solutions listed above. In addition, samples ranging between 0.1–5.0 mM for GdCl_3_ and 1.0–50.0 mM for TmCl_3_ were prepared with NaCl at 300 mOsm/L. To test the effect of protein content, the same series of samples of TmCl_3_ and GdCl_3_ were prepared with 90 μM of albumin, using a 300 μM stock solution of albumin and the TmCl_3_ and GdCl_3_ stock solutions listed above.

Rat liver and kidney tissues were excised and placed into 10% neutral buffered formalin and stored at 4°C before they were used for each tissue sample preparation. Rat kidney and liver tissue samples weighting 100 mg were tested, and the density of each tissue was assumed to be 1 g/mL to determine the volume of the tissue. Tissue samples that weighed 50, 100, 150, 200 and 250 gm were also tested. A stock solution of each lanthanide ion or gadodiamide was then added to a tissue sample, and diluted with distilled water, to create a range of tissue samples that ranged between 0.1–5.0 mM for GdCl_3_ and gadodiamide, 0.5–10 mM for DyCl_3_, and 1.0–50.0 mM for TmCl_3_, EuCl_3_, and YbCl_3_. A final volume of 5 mL was used for tissue samples with a concentration less that 5 mM, and tissue samples with a greater concentration were prepared with a final volume of 1 mL. The tissue samples were allowed to stand at room temperature for 30 min before lyophilization. Upon complete removal of water, the samples were suspended in 1 mL or 5 mL of conc. nitric acid for 3–5 h to allow for complete decomposition of the biological material.

A volume of 200 μL of each sample was added to a clean 3 mm NMR tube, and this narrow NMR tube was then placed in a 5 mm NMR tube that contained 300 μL of concentrated nitric acid as a reference solution. The heights of the sample volumes in the co-axial tubes exceeded the height of the NMR transceiver coil. Thus the sample approximated an infinite cylinder parallel to the static magnetic field of the NMR spectrometer, which provided the greatest BMS shift (*i.e.*, s = 1/3 in Equation (1)). Samples were equilibrated at each temperature before acquiring spectra. A one-dimensional NMR spectrum was acquired for each sample, with a 2.19 s repetition time; 7 μsec excitation pulse; 12.5 ppm spectral width; 16,384 acquired data points; 0.114 Hz/point spectra resolution after zero-filling; and four repetitions with phase cycling. A deuterium lock was not used, because adding deuterium to the sample may have changed the BMS effect. Sample spinning was not used and shimming was not required, because the resulting lineshapes were sufficiently narrow and symmetric for accurately measuring the BMS shifts in each spectrum. The separation between the two spectral peaks was recorded in Hz to ensure measurement precision, and the Larmor frequency was used to convert this measurement to units of ppm. NMR spectra were recorded using a 300 MHz (7 T) Varian Unity+ spectrometer with an inverse tranceiver probe, and a 600 MHz (14 T) Varian Inova spectrometer with an inverse tranceiver cryoprobe. Measurements were acquired at 22.0 °C, except for some measurements performed at temperatures as high as 67.0 °C, using the calibrated temperature unit of the spectrometer.

For each tissue study, three samples at each concentration of lanthanide ion and gadodiamide were prepared and measured with BMS NMR spectroscopy to ensure reproducibility. The other studies were performed with only one sample per concentration of lanthanide ion and gadodiamide. The BMS shift measured for each tissue sample was converted to a concentration of lanthanide ion and gadodiamide using each calibration determined with lanthanide ion and gadodiamide solutions. The calculated concentrations were compared with the concentrations used during preparation of the sample for BMS NMR spectroscopic analyses.

## 3. Results and Discussion

A variety of methods for preparing samples were initially tested, including combinations of burning tissue samples to ash in a 600 °C furnace, homogenizing tissue samples to a paste, and high-speed centrifugation that have been previously used to extract metals from tissues [24–26]. These methods provided disappointing 8%–10% yields of recovered lanthanide ion in tissues. Therefore, these other methods were abandoned when a simplistic treatment with nitric acid was found to provide excellent yields [27]. The nitric acid completely digested tissues within 20 min, resulting in clear solution with a yellow color.

**Figure 1.**
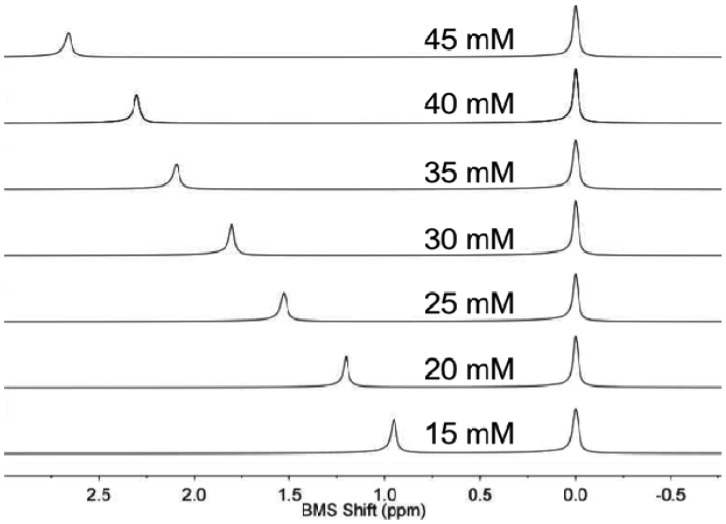
NMR spectra of coaxial samples of Tm(III) ion in nitric acid (left peak) and nitric acid without Tm(III) (right peak) were rapidly acquired and easily analyzed to measure the BMS shift of each sample.

NMR spectroscopy of the sample and control solution in co-axial tubes generated outstanding spectral results (Figure 1). An initial study demonstrated that the BMS shift was invariant within one minute of inserting the sample in the magnet at 22.0 °C, indicating that the sample equilibrated from room temperature to 22.0 °C within one minute. The acquisition of the NMR spectrum required 8.76 s. Data processing and analysis required less than one minute. Therefore, the total time to analyze one sample was approximately two minutes. Thirty samples were routinely analyzed within one hour.

**Figure 2.**
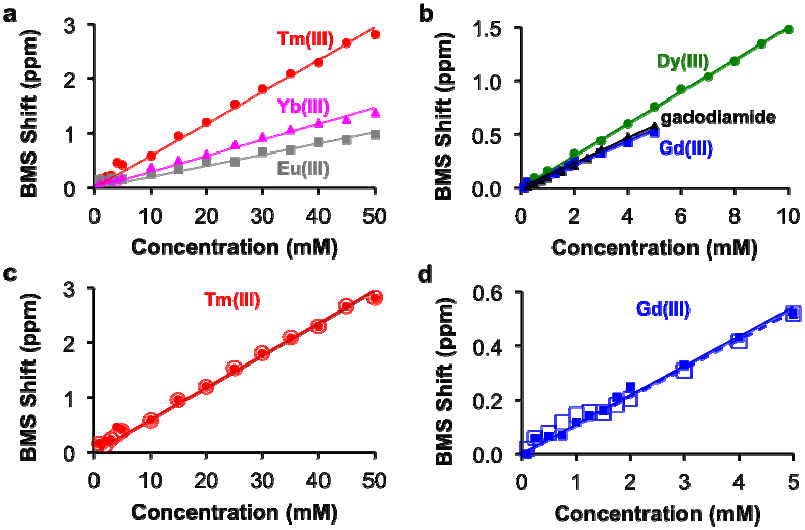
Correlation of BMS shift and lanthanide ion concentration. (**a**,**b**) The correlation of BMS shift and concentration for each lanthanide ion or gadodiamide at 7 T magnetic field strength showed outstanding linearity with R^2^ > 0.98. (**c**,**d**) The concentration dependence of BMS shift at 7 T (filled symbols) and 14 T (open symbols) for Tm(III) and Gd(III) showed that the calibration was independent of magnetic field strength.

The concentrations of each lanthanide ion and gadodiamide in nitric acid were linearly correlated with BMS shifts acquired at 7T magnetic field strength. (Figure 2a,b). The calibrations with Gd(III) and gadodiamide were identical, indicating that the nitric acid digested the chelator, or the chelator had negligible effect on the BMS shift. Gd(III), gadodiamide, and Dy(III) were analyzed using concentration ranges that were lower than the ranges used for the other lanthanide ions, because very high concentrations for Gd(III) and Dy(III) caused rapid T2 relaxation that led to substantial line broadening in the NMR spectrum. These correlations showed outstanding linearity with R^2^ correlation coefficients greater than 0.98. The slope of each calibration was used to determine *μ_eff_* for each lanthanide ion, which agreed with previously published results (Table 1; Equation (2)). This agreement validated that the concentration dependence of the measured chemical shifts arose from a BMS shift:

(2) $$\mu_{\text{eff}}^{2}=\frac{\text{T}}{519.3}\frac{\Delta_{\chi }}{\left[Ln\right]}=\frac{\text{T}}{519.3}(\text{slope})$$

**Figure 3.**
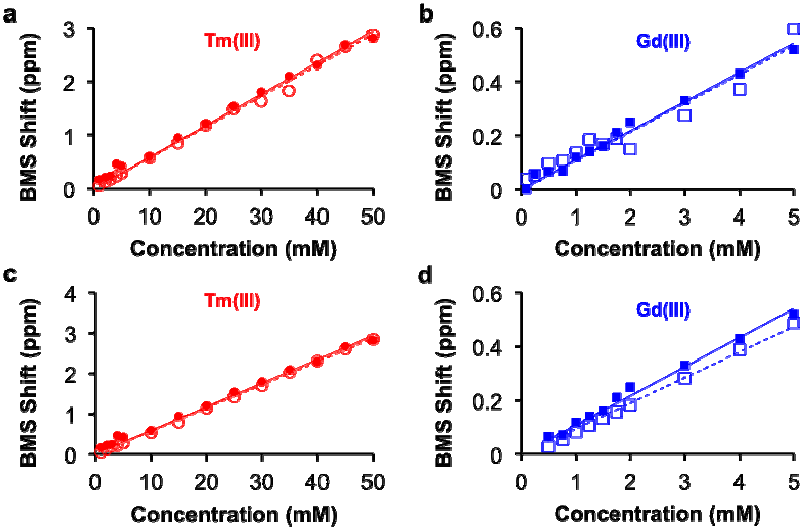
Effect of sample conditions on the concentration-BMS shift calibration. The calibrations of (**a**) Tm(III) and (**b**) Gd(III) without (filled symbols) and with 300 mOsm/L NaCl (open symbols) showed that salt had a neglible effect on the concentration-dependent BMS shift. The calibrations of (**c**) Tm(III) and (**d**) Gd(III) without (filled symbols) and with 90 μM albumin (open symbols) showed that proteins had a neglible effect on the concentration-dependent BMS shift.

This study with Tm(III) and Gd(III) samples at different concentrations was repeated at 14 T magnetic field strength, which generated the same calibration (Figure 2c,d). This result indicated that NMR spectrometers at any field strength may be used to measure lanthanide ion concentration via BMS shift measurements. The calibration of BMS shift with the concentration of Tm(III) or Gd(III) was not affected by the addition of 300 mOsm/L salt or 90 μM protein (Figure 3). In addition, samples of 15 mM Tm(III) and 5 mM Gd(III) that had varying concentrations of salt generated identical BMS shifts (data not shown), which further verified that salt had no effect on the BMS measurement. These results indicated that estimates of lanthanide ion concentrations via BMS shift measurements with sample preparation using nitric acid could be translated to tissue samples that have high salt and protein contents.

**Figure 4.**
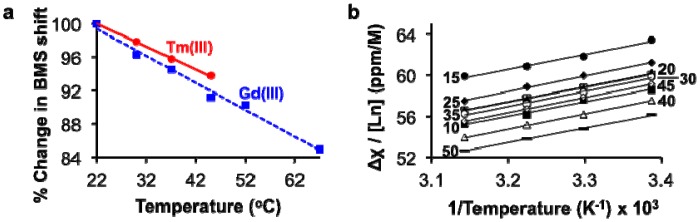
Effect of temperature on the concentration-BMS shift calibration. (**a**) The greatest BMS shifts were observed at lowest temperatures. (**b**) The BMS shift, Δ_χ_, had a linear dependence on lanthanide ion concentration and inverse temperature, as predicted by theory (Equation (3)). Each line is labeled with is concentration in mM, with labels for data with solid symbols on the left and labels for data with open symbols on the right.

The BMS shifts of Tm(III) and Gd(III) decreased with increasing temperature, as predicted by Equation (1) (Figure 4a). This result indicated that analyses of lanthanide ion concentrations should be conducted at lower temperatures to improve measurement precision. We elected to perform measurements at 22 °C, the lowest temperature tested in our studies, to avoid delays required to equilibrate the sample at lower temperatures. The values of *μ_eff_* for Tm(III) and Gd(III) were estimated based on the dependence of the BMS shift on concentration and temperature (Table 1; Equation (3); Figure 4b). Once again, these estimates of *μ_eff_* agreed with published results, which further validated that the BMS shift was the primary mechanism responsible for the differences in chemical shifts observed using NMR spectroscopy:

(3) $$\mu_{\text{eff}}^{2}=\frac{1}{519.3}\frac{\text{T}\Delta_{\chi }}{\left[Ln\right]}=\frac{1}{519.3}(\text{slope})$$

**Figure 5.**
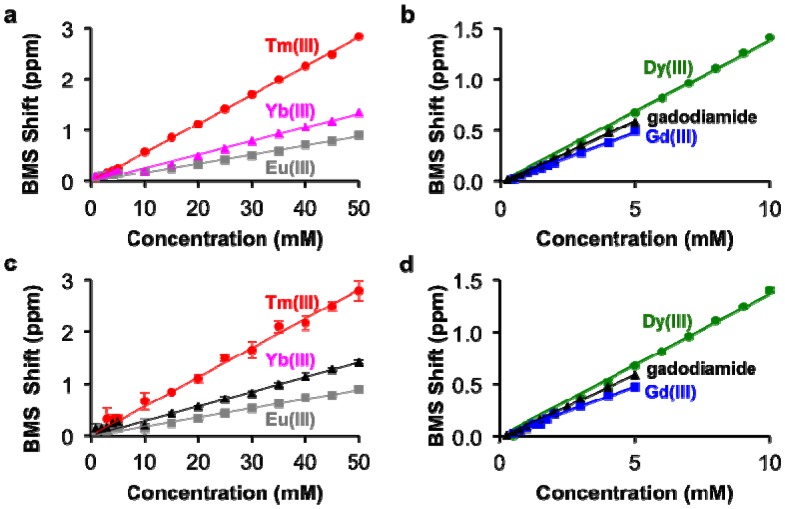
Correlation of BMS shift and concentration of lanthanide ion or gadodiamide in (**a**,**b**) rat kidney tissues and (**c**,**d**) rat liver tissues showed outstanding linearity with R^2^ > 0.97.

The concentrations of each lanthanide ion and gadodiamide in kidney and liver tissues treated with nitric acid were linearly correlated with BMS shifts acquired at 7T magnetic field strength. (Figure 5). These relationships had *R*^2^ correlation coefficients greater than 0.96. These outstanding linear correlations with a 0 y-intercept indicated that endogenous metals such as iron had negligible effects on the BMS measurements. Furthermore, the average standard deviations from triplicate evaluations of each sample were less than 10%, and the great majority of standard deviations were much smaller than 10%, as evidenced by many error bars that are smaller than the size of the data symbol in Figure 5. These results demonstrated that the concentration of lanthanide ion or gadodiamide in tissues was measured with outstanding precision. In addition, the correlations for Gd(III) and gadodiamide were the same, which once again indicated that nitric acid digested the chelator during tissue digestion, or the chelator had negligible effect on the BMS shift. Identical results were obtained using samples that ranged from 50 to 250 mg of tissue, indicating that the tissue volume did not influence the measurement precision (data not shown), which further verified that protein content within the processed solution did not influence the measurement of lanthanide ion concentration with BMS NMR spectroscopy.

**Figure 6.**
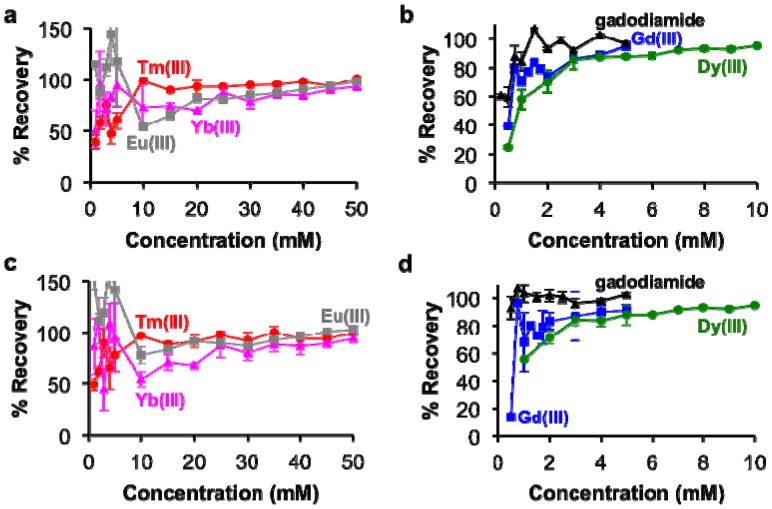
The recovery of lanthanide ions from (**a**,**b**) rat kidney tissues and (**c**,**d**) rat liver tissues. (a,c) At least 80% recovery was obtained for samples with ≥ 25 mM Tm(III), Yb(III), and Eu(III). (b,d) At in 85% recovery was obtained for samples with ≥ 3 mM Gd(III), gadodiamide, and Dy(III).

The percent recovery of lanthanide ions from the tissue samples was determined by using the calibrations in Figure 2 to estimate the ion concentrations from the BMS shifts of the tissue samples (Figure 6). These results showed greater than 85% recovery with samples containing at least 3 mM with Gd(III), gadodiamide, or Dy(III), and greater than 80% recovery with samples containing at least 25 mM of Tm(III), Yb(III), and Eu(III). However, the recovery was less accurate for more dilute samples. This result reflects the difficulty in sample handling, which affects dilute samples to a greater extend than concentrated samples. In particular, the development of our methodology showed that care needs to be taken to handle nitric acid without loss due to evaporation, which can be substantial over time and especially during outdoor transport in a southwest desert environment.

These results established a protocol for measuring the lanthanide ion concentration in *ex vivo* tissues. Nitric acid can be used to digest any tissue or lanthanide chelate, which allows this method to be applied to many biomedical studies. NMR spectrometers at any magnetic field strength are commonly available at many research institutions for a nominal usage fee. Many samples can be quickly analyzed with this method. As an additional benefit, the completely digested tissues are not biohazardous, which facilitates waste handling (however, care should be taken to properly dispose of samples with lanthanide ions).

Our results show that lanthanide ion content can be measured in *ex vivo* tissues with outstanding precision. In addition, accurate measurements can be made with concentrations ≥ 25 mM for Tm(III), Eu(III) or Yb(III), and ≥ 3 mM for Gd(III), gadodiamide, or Dy(III). Unfortunately, T1 and T2 MRI contrast agents typically accumulate in tissues at 10 μM to 1 mM concentrations, and paramagnetic CEST agents likely accumulate in tissues at concentrations less than 10 mM. Therefore, the current concentration thresholds for accurate measurements are too high to apply this method for most biodistribution studies of MRI contrast agents. The lower yields at low concentrations are attributed to the loss of lanthanide ions or chelates during tissue processing. Therefore, improved tissue handling methods are warranted to facilitate this methodology. For example, extreme care is needed for handling and processing lanthanide chelates for optical imaging studies, and these careful handling methods may be adopted for measuring BMS shifts with NMR spectroscopy [28]. As an alternative, lyophilization of a large volume of tissue may be digested with a small volume of nitric acid, which would effectively concentrate the lanthanide ion in the acidic solution used for BMS NMR measurements. The volume of tissue that can be digested by 1 or 5 mL of nitric oxide (the volumes of acid used in this study) would need to be tested for each tissue type to assess the improvement in detection sensitivity by using this approach. Also, the influence of concentrating endogenous metals such as iron would also need to be assessed.

Other biological and non-biological samples besides *ex vivo* tissues also require testing for lanthanide concentrations. For example, biosorption of lanthanide metals has been monitoried in microbial species [29], plants [30], and biomass [31] to assess environmental quality and bioremediation. The concentrations of lanthanide metals are also assessed in soils [32,33] and stream waters [34,35], especially near mining sites and industrial complexes. These environmental assessments are likely to continue, considering the ∼10%/year increase in lanthanide ore production to meet rising demands for glass polishing, catalysts, phosphors, magnets and electronic products [36]. As with the analyses of tissues in this report, the application of acid treatment and BMS shifts for measuring lanthanide concentrations in other samples will require consideration for the minimum detection level and/or consideration for concentrating the sample to meet the minimum detection level for accurate and precise measurements of lanthanide metal concentrations.

## 4. Conclusions/Outlook

The BMS shift was successfully used to measure the concentration of lanthanide ions and a MRI contrast agent, gadodiamide, in solution and within *ex vivo* tissue samples. Key steps in this process included the use of nitric acid to completely digest tissues, and the use of coaxial tubes for simultaneous NMR spectroscopy of experimental and control samples. The concentration dependence of the measured chemical shifts was shown to be due to the BMS shift. Although the methodology had outstanding precision, concentration measurements were accurate only for samples with higher concentrations. Improvements to sample handling during tissue processing are warranted to improve the accuracy of measuring lanthanide ion concentrations of more dilute samples.

## Figures and Tables

**Table 1.** Estimates of *μ_eff_* from BMS shifts.

**Lanthanide Ion**	***μ****_eff_* **(Figure 2a,b)**	***μ****_eff_* **(Figure 4a)**	***μ****_eff_* **([22])**
Dy(III)	10.38	---	10.6
Gd(III)	7.86	8.10	7.94
Tm(III)	6.56	7.54	7.6
Yb(III)	3.81	---	4.5
Eu(III)	3.18	---	3.40–3.51
